# Supplementary material for: Assessing Negative Welfare Measures for Wild Invertebrates: The Case for Octopuses
Source: Animals (Basel). 2023 Sep 26;13(19):3021. doi: 10.3390/ani13193021 (PMC10571587; doi:10.3390/ani13193021)
Supplement: Supplementary file 1 [file animals-13-03021-s001.zip › animals-2600089-supplementary.pdf]

Supplementary Materials

Table S1. Body patterns and components definitions.

Body patterns and/or components	Definitions
Blotch	The chromatic background of the octopus is in variations of reddish brown (light or dark), blotches and spots present on the mantle and sometimes extending to the beginning of the arms. Pale or dark arms, the eyes have dark bars (eyebar) and the textural component is smooth.
Mottle	The chromatic background of the octopus is in variations of reddish brown, with alternating bars on all the arms, a blue-green sheen around the eyes, a white V on the middle of the head. The texture may be smooth, rugose, or textured, usually accompanied by an eyebar.
Uniform dark	The animal is found with dark chromatic components all over its body background, with smooth or rugose textural components. Sometimes it is accompanied by an eyebar.
Uniform light	The entire body of the animal is in a white hue. This pattern accompanies the smooth textural component and can occur either in motion or action. It can accompany an eyebar and also the ventral part of the arms in reticulated red.
Light brown	Most of the animal's body is in a uniform light brown pattern, usually associated with a smooth textural pattern.
Deimantic display	The octopus is mostly pale, with darkness around the head - "hood" - usually accompanied by an eyebar and a pale hood over the eyes; Arms curved in arc and web spread.
Tricolor longitudinal stripes	Longitudinal tricolor stripes on the mantle towards the arms, one light brown, one uniform dark and one uniform light more positioned on the right side of the body. Smooth textural pattern and dark suckers.
White longitudinal stripe	White longitudinal stripe on the mantle going towards the arms, surrounded by a pattern of uniform dark stripes on both sides of the body. Generally the animal is in smooth texture and with darkened suckers in this pattern.

Half and half	Unilateral pattern with dark components on one side of the body and light components on the other side and the texture is smooth or rugose.
Half and half blotch	Unilateral pattern with a distinct longitudinal line dividing them. The blotch pattern consists of white circles on a brown background. The texture is smooth. This pattern can occur when the animal is standing still or in motion.

Table S2. Arm loss table in octopuses found in our sampling.

Octopus	Context	No	1L	2L	3L	4L	1R	2R	3R	4R	Arm loss	How many
41	Agonistic interaction		1				1	1			Yes	3
43	Agonistic interaction		1					1			Yes	2
15	Fish	-	-	-	-	-	-	-	-	-	Not visible	-
16	Fish						1				Yes	1
44	Fish		1				1				Yes	2
48	Fish	-	-	-	-	-	-	-	-	-	Not visible	-
49	Fish	1									No	-
53	Fish	-	-	-	-	-	-	-	-	-	Not visible	-
47	Fish	-	-	-	-	-	-	-	-	-	Not visible	-
57	Fish	-	-	-	-	-	-	-	-	-	Not visible	-
1	Fishing			1	1			1			Yes	3
2	Fishing				1		1	1	1		Yes	4
3	Fishing					1				1	Yes	2
4	Fishing			1	1	1	1				Yes	4
5	Fishing					1			1		Yes	2
6	Fishing	-	-	-	-	-	-	-	-	-	Not visible	-
7	Fishing			1	1						Yes	2
8	Fishing			1			1		1		Yes	3
9	Fishing						1	1	1		Yes	3

10	Fishing		1								Yes	1
11	Fishing							1			Yes	1
12	Fishing			1							Yes	1
13	Fishing							1			Yes	1
18	Fishing									1	Yes	1
28	Fishing					1					Yes	1
29	Fishing								1		Yes	1
30	Fishing	1									Yes	1
31	Fishing		1				1		1		Yes	3
32	Fishing							1			Yes	1
33	Fishing	-	-	-	-	-	-	-	-	-	Not visible	-
34	Fishing						1				Yes	1
55	Fishing		1								Yes	1
17	Fishing	-	-	-	-	-	-	-	-	-	Not visible	-
42	Fishing		1								Yes	1
19	Foraging		1								Yes	1
22	Foraging		1								Yes	1
23	Foraging		1								Yes	1
25	Foraging		1								Yes	1
35	Foraging		1								Yes	1
46	Foraging		1								Yes	1
50	Foraging	1									No	
54	Foraging		1								Yes	1
21	Foraging		1				1	1			Yes	3
40	Foraging						1				Yes	1
45	Foraging	-	-	-	-	-	-	-	-	-	Not visible	-
52	Foraging		1								Yes	1

56	Foraging						1				Yes	1
24	In den		1								Yes	1
26	In den		1								Yes	1
27	In den	-	-	-	-	-	-	-	-	-	Not visible	-
36	In den	-	-	-	-	-	-	-	-	-	Not visible	-
37	In den	-	-	-	-	-	-	-	-	-	Not visible	-
38	In den	-	-	-	-	-	-	-	-	-	Not visible	-
39	In den	-	-	-	-	-	-	-	-	-	Not visible	-
51	Senescence	1									No	-
14	Predation	-	-	-	-	-	-	-	-	-	Not visible	-
20	Predation	-	-	-	-	-	-	-	-	-	Not visible	-

Table S3. Residuals table of chi-square tests of body patterns by context.

Context	Blotch	DD	HH	HH Blotch	Brown	Mottle	Uniform light	TLS	Uniform dark	WLS
Interaction with fishermen	2,94218271	-0,63427033	0,72192954	-1,41827157	1,02096255	-2,78545218	0,10959161	0,21784133	0,22199462	-0,8969937
Interaction (agonistic) with other octopus	0,035033	-0,214423	-0,371391	-0,479463	-0,525226	-1,050451	-0,567309	2,944873	0,308232	2,994487
Interaction with fishes	-1,37727271	-0,4152274	-0,71919495	4,45668812	-1,01709526	1,40698177	-1,09858844	-0,58722022	-1,05882987	1,11571842
Predation	-0,69480833	-0,15161961	-0,26261287	-0,33903175	-0,37139068	0,60350985	-0,40114778	-0,21442251	1,04238481	-0.21442251
In den	-1.38961667	-0.30323922	-0.52522573	-0.67806350	0.60350985	1.88016530	0.44412790	-0.42884501	-0.38852525	-0.42884501
Foraging	-1.43652065	1.48573854	0.27713265	-1.12444111	-0.41991938	1.59569366	0.17278658	-0.71115900	0.47429346	-0.71115900
Senescence	-0,49130368	-0,10721125	-0,18569534	-0,23973165	-0,26261287	-0,52522573	3,24176359	-0,1516196	-0,42884501	-0,1516196

Table S4. Residuals table of chi-square tests of injuries by context.

Context	Arms	Between_arms	Head_and_eyes	Mantle	Membrane	Scukers	No	Not_visible
Interaction with fishermen	17.759.569	-0.3261962	-0.2663381	0.2731996	0.9423445	13.326.764	-0.2879388	-15.304.862
Interaction (agonistic) with other octopus	-0.6432675	-0.6432675	13.787.175	35.073.069	-0.2144225	-0.3032392	-12.685.407	-0.8022956
Interaction with fishes	-12.456.822	-0.4429092	-10.170.953	-0.6510180	-0.4152274	-0.5872202	0.3930429	23.082.632
Predation	-0.4548588	-0.4548588	-0.3713907	14.857.385	-0.1516196	-0.2144225	-0.8969937	11.954.003
In den	0.1895245	0.1895245	32.960.923	-10.057.307	-0.3032392	-0.4288450	-12.365.699	0.6280917
Foraging	-0.8457281	11.428.758	-12.317.635	-10.682.291	-0.5028654	-0.7111590	17.309.042	-0.8185964
Senescence	-0.3216338	-0.3216338	-0.2626129	-0.3555795	-0.1072113	-0.1516196	20.916.991	20.916.991

Table S5. Residuals table of chi-square tests of irregular chromatophore expressions by context.

Context	No	Not visible	Yes
Interaction with fishermen	-0.5527161	-26.151.636	27.726.280
Interaction (agonistic) with other octopus	-0.6948564	25.092.459	-11.141.720
Interaction with fishes	0.5825586	17.925.924	-21.575.849
Predation	10.173.580	-0.6251437	-0.7878386
In den	-0.9826754	27.487.935	-0.9410294
Foraging	0.9481209	-11.087.524	-0.3167230
Senescence	-0.7030312	18.201.784	-0.5570860

Table S6. Residuals table of chi-square tests of abnormal body positions by context.

Context	No	Not visible	Yes
Interaction with fishermen	-25.532.426	20.213.637	31.045.622
Interaction (agonistic) with other octopus	0.8164966	-0.8304548	-0.8022956
Interaction with fishes	15.811.388	-16.081.688	-15.536.387
Predation	-0.2886751	-0.5872202	11.954.003
In den	-0.5773503	22.314.368	-11.346.173

Foraging	19.148.542	-19.475.891	-18.815.499
Senescence	0.4082483	-0.4152274	-0.4011478

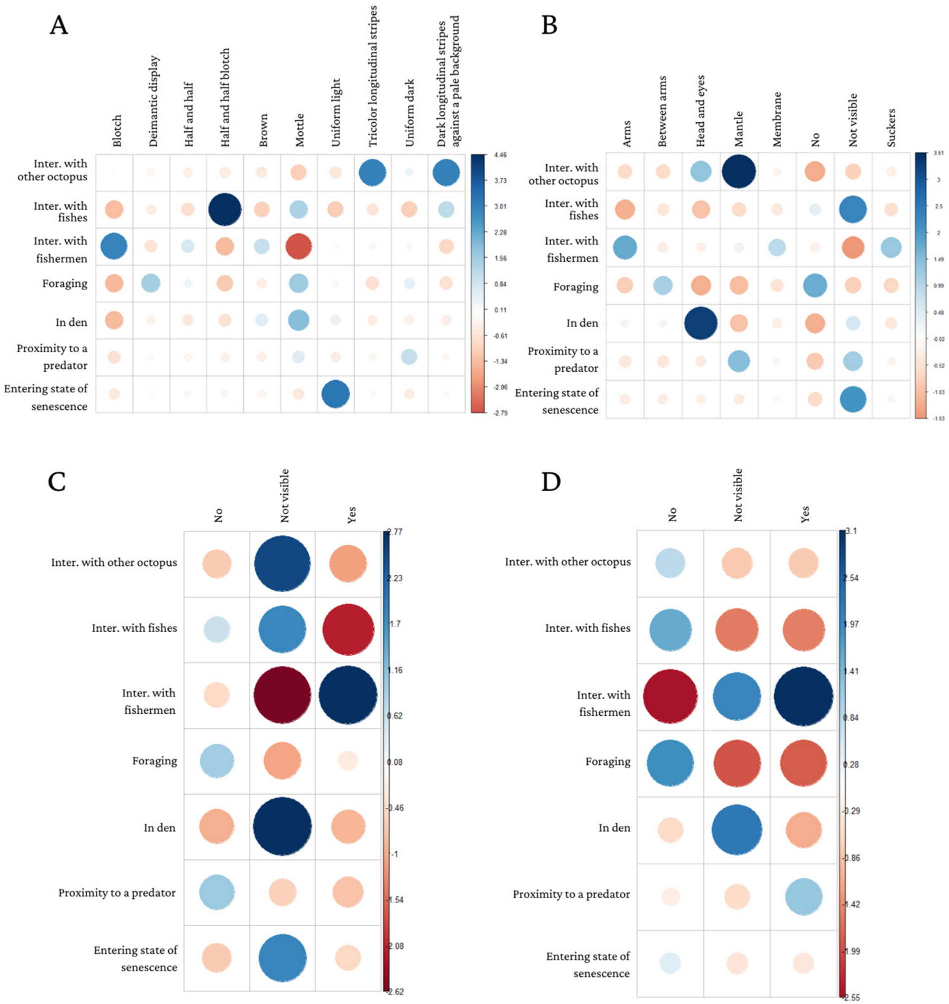

Figure S1. Correlation matrix between the variables based on chi-square tests. (A) Body pattern, (B) injuries, (C) irregular chromatophore expression, and (D) abnormal motor coordination. Positive correlations are in blue; negative correlations are in red.
